# The adverse impact of excessive internet use during the COVID-19 pandemic on adolescents' coping skills: A case study in Hanoi, Vietnam 2021

**DOI:** 10.3389/fpubh.2022.983153

**Published:** 2022-09-15

**Authors:** Quyen Thi Tu Bui, Chi Thi Lan Pham, Anh Ha Le, Duy Quang Pham

**Affiliations:** ^1^Faculty of Fundamental Science, Hanoi University of Public Health, Hanoi, Vietnam; ^2^Faculty of Sciences, University of British Columbia, Vancouver, BC, Canada; ^3^Hanoi Amsterdam High School, Hanoi, Vietnam

**Keywords:** COVID-19, children, adolescent, mental health, pandemic effects

## Abstract

**Introduction:**

The COVID-19 pandemic has created significant stressors in Vietnamese adolescents' lives. Coping skills play important roles in helping adolescents contend with stress. This study aimed to evaluate adolescents' coping skills during the COVID-19 pandemic and examine how those skills are impacted by excessive internet use during this pandemic.

**Methods:**

The study used respondent-driven sampling and Google online survey forms to collect data. The study sample included 5,315 high school students aged 11- 17 years in Hanoi's rural and urban areas. The Kid Coping Scale was applied to examine adolescents' coping, and the coping score was compared among adolescents with different levels of internet use.

**Results:**

The average coping score measured by Kid Coping Scale was 20.40 (std = 2.13). About half of adolescents often “avoid the problem or the area where it happened” when experiencing a hard time. One-third of adolescents often stopped thinking about the problem they faced. More than one-fourth of respondents stayed online for at least 8 h per day. The online time for learning/other activities showed a reverse dose-response relationship with the coping score; the longer the internet use duration, the lower the coping score.

**Conclusion:**

The mean score of coping of Hanoi adolescents was moderate. Internet use has an adverse impact on their coping skills.

## Introduction

Coronavirus disease 2019 (COVID19), which was declared a pandemic on March 11, 2020 (1), is currently causing thousands of deaths worldwide. Because of this situation, social confinement for the entire population, including adolescents, became mandatory in many countries.

Adolescents are a vulnerable population undergoing a challenging transition period (2). COVID-19 detention has impacted adolescents' physical, mental, and emotional well-being (3). Lockdown due to the COVID-19 pandemic has obliged school children to stay at home, have e-classes from home, and increase the time of access to the internet and social media (4). School children see their family members too much while having too little interaction with friends (5). Apart from that, all plans are canceled or postponed quite often. Children worry about the economic future of their family and country, have felt less secure than in the past (4), and are uncertain about the future. All these things can make them stressed during a challenging time (6–9). The pandemic has affected children at different levels – health, social, family, and individual (10). They worried about their family, friends, and physical health; they also felt unfocused and anxious and had negative thoughts during the pandemic (11). The physical and mental impact of the COVID-19 epidemic on children and adolescents is a matter of fundamental importance both for governments and families (4).

One of the most potent risk factors for psychopathology during childhood and adolescence is exposure to acute and chronic stressful events and adversity. However, not all children and adolescents who face stress and adversity develop symptoms of psychopathology, raising the question of why some children and adolescents suffer while others are resilient. The ability to cope with stressful events and circumstances and regulate emotions in various situations may play an essential role in developing resilience and reducing the risk of psychopathology during childhood and adolescence (12, 13).

Coping is a complex concept that can protect against or increase the risk of adverse mental health outcomes during stressful life experiences. Coping was defined as “constantly changing cognitive and behavioral efforts to manage specific external and internal demands that are appraised as taxing or exceeding the person's resources” (14). Coping is important in considering how stressors affect children and adolescents because it emphasizes a child's active role in the transactional process of dealing with stressful situations in their lives while also bringing reflections about one's future development (13). The scope of coping has broadened with a growing emphasis on coping as the regulation of a broader range of functions, including emotion, behavior, cognition, physiology, and the environment, in response to stress (15).

During the COVID-19 pandemic, children are not allowed to go to school and must continue to study at home. This increases their internet usage during the day. In addition to using cyberspace for their studies, students access it for many other purposes, such as reading news, surfing websites, and joining social networks. Long internet use duration has been demonstrated to affect children's health, social competence, and behavioral competence (16). Understanding how the youth cope with such significant life upheavals will help ensure that youth support activities are appropriately targeted and successful. However, the literature on children's coping strategies during the COVID-19 pandemic is still limited. In October 2021, many new cases and deaths due to COVID-19 continued to be reported in Vietnam (17), and schools in many provinces (including Hanoi) were closed. Hanoi have been applied online teaching and learning.

This study, therefore, was conducted to investigate coping skills among adolescents in Hanoi and explore the association between the average time of their internet use per day and their coping skills.

## Materials and methods

### Study design and setting

This study employed a cross-sectional design conducted in Hanoi Capital, Vietnam. Hanoi consists of 12 urban districts, one district-leveled town, and 17 rural districts (18).

### Study participants

The inclusion criteria for participants were children aged 11–17 years old, living in Hanoi, and accessing the internet to complete the form. No exclusion criteria were applied in the study.

### Sample size and sampling method

The sample size was calculated based on a single population formula with absolute precision. We had no information about the proportion of children with coping issues in Vietnam, so we chose *p* = 0.5 to maximize the sample size and a small d of 0.03, 95% confidence level at z = 1.96, producing a sample size of 1,068 children. Since we want to provide estimation separately for male/female and urban/rural and estimated non-response rate of 25%, the estimated sample size was 5,340.

The respondent-driven sampling method was used, and data were collected using Google online survey forms. We started with a group of 100 core members selected representatives of different age from 11 to 17, male and female (50% was male and 50% was female), rural and urban (students was randomly invited from five rural districts and five urban districts). Each core member was asked to send the forms to five friends to complete and ask each of those friends to continue forwarding the link to the next five friends. The participants can forward the link through zalo, Facebook, or email. The study sampled 5,315 students aged 11–17 years attending some secondary and high schools in Hanoi's rural/urban areas. The secondary school students accounted for 55.94% of all respondents; the rest were high school students.

### Data collection

Data was collected through a monkey survey in Google Forms. The electronic questionnaire's content and interface were designed and tested so that the audience could answer the survey on such media as smartphones, tablets, and computers. Respondents completed the online survey between October and December 2021. The survey included demographic factors, hardships during the COVID-19 pandemic, family care, family pressure, and coping strategies. The Kid Coping Scale (KCS) was applied to examine their coping skills.

### Measurements

#### Coping scale

The studies based on the research work of Maybery et al. (19) on coping strategies for stressful situations (Kid Coping Scale-KCS) examined three main coping strategies: (1) Problem-focused coping (try to think the different way to solve the problem*/try the best to make things better/try hard to fix the problem/say sorry if it is your fault); (2)* Emotion-focused coping *(Distracting yourself from thinking about the problem/ avoid the problem or the area where it happened/ did something else to stop thinking about the problem); (3)* Seeking social support *(thought about what others might do/sought help from others)*. The KCS applied the Likert scale (with three levels: never, sometimes, and always).

Up to now, the Kid Coping Scale was not available in Vietnamese. The research team applied the following process to translate the tool: (1) two people translated the questionnaire into Vietnamese and compared their translation to make changes when necessary (2) back translation: the Vietnamese version was translated into English by a third person to make sure no contents of the questionnaire was lost during translation (3) pretest the translated Vietnamese version: 10 high school students were selected for the pretest, they provided comment on words that don't quite translate well, were ambiguous; and (4) finalizing translation. Internal consistency in the current sample was 0.81 for problem-solving coping, 0.79 for avoidant coping, and 0.90 for social support-seeking coping.

The scores for each component's total coping skills were calculated by taking all items/items in each element. The KCS had nine items, which entity would have a score range from 1 (never) to 3 (always), so the minimum score was nine and the maximum score was 27. The higher score represents a greater active/passive coping tendency. Besides that, the adolescent who has an issue with coping if they answered at least 1 question as never in the domain (emotion-focused coping, problem-focused coping, and seeking social support).

#### Internet use

Measuring the daily average online time of an adolescent. During Covid time, all the secondary high school children study online for an average of 3.5 h in the morning, and Jason et al. mentioned that the average time for kids using the internet was 8 h during Covid time (20). We consider more than 8 h was high, but we also want to look at a lower cut-off point. Internet use was categorized into four groups: <4 h, 4–6 h, 6–8 h, and 8 h and more.

### Family care and family pressure

Family care and family pressure were measured by the Global School-based Student Health tool (21). This tool applied the Likert scale (with five levels, namely never, rarely, sometimes, often, and always). It consisted of seven items regarding the frequencies at which parents or guardians did during the past 30 days (four items for family care and three items for family pressure): (1) the parent/guardian checked if their child did homework, (2) the parent/guardian understood their child's problems and worries, (3) the parent/guardian knew what their child was doing in his/her free time, (4) the parent/guardian gave advice and guidance to their child, (5) the parent/guardian expected too much of their child, (6) the parent/guardian did not respect their child as a person, and (7) the respondent was involved in a physical fight during the past 12 months. The sum of the item score was used as the scale for family care and family pressure.

Other information included demographic information (gender, age), grade, and the person with whom the child lives (parents, a single parent, and neither mother/father).

### Data analysis

In this study, research data were expressed as frequencies and percentages. Frequencies and means were used to identify the rates of coping strategies children used. Univariate and multiple linear regression models were applied to determine the relationship between independent factors and the coping score (dependent variable). We had a conceptual framework for the original study for all the related factors of children's psychological status like coping skills. The questionnaire (for related factors) was developed based on literature and the variables selected for the multiple regression model were chosen based on that. The univariate analysis was performed, and the results of these relations with a *p* < 0.2 were included in the multivariate linear regression. A *p*-value <0.05 was regarded as statistically significant. Statistical analyses were performed using STATA version 17.0.

### Ethics

Ethical clearance, including confidentiality of the participants' consents and information, was approved by the Human Research Ethics Committee at The first author's institution with No. 382/2021/YTCC-HD3. No sensitive data that could identify the participants was collected. Informed consent was described in the instrument, with mandatory acceptance to proceed. There was also a contact for further clarification.

As the survey collected data through Google form, we anticipated it would be challenging to obtain parental and children's consent forms. Also, the 45 CFR § 46.104 - Exempt research stated that “*research subject to subpart D involving educational tests or the observation of public behavior when the investigator(s) do not participate in the activities being observed*” may have exempt of parental ethnics (22). We had sought the permission of the IRB the waive parental consent forms.

## Results

Table 1 shows the sociodemographic characteristics of the study participants, including 5,315 children aged 11 to 17 years, with 2,177 boys (41.0%) and 3,138 girls (59%). Ninety-two percent of the children reported living with their parents, 7.6% living under one roof with a single parent, and 0.4% living alone. About 53% of the participants are from the rural area of Ha Noi. During the COVID-19 pandemic, about 27.3% of the participants lived in families that experienced difficulty in buying food. One-fourth of the children had at least one parent who became jobless due to COVID-19. About 12% had experienced domestic family violence during this pandemic. Children surfing the internet for at least 6 h a day accounted for 50% of all participants.

**Table 1:** Characteristics of the study sample.

**Characteristics**	**Male; *n* (%)**	**Female; *n* (%)**	**Total; *n* (%)**
	***n* = 2,177 (41.0)**	***n* = 3,138 (59.0)**	***n* = 5,315**
Age			
Aged 11	138 (6.3)	195 (6.2)	333 (6.3)
Aged 12	147 (6.7)	221 (7.0)	368 (6.9)
Aged 13	232 (10.7)	260 (8.3)	492 (9.3)
Aged 14	305 (14.0)	342 (10.9)	647 (12.2)
Aged 15	609 (28.0)	877 (27.9)	1,486 (28.0)
Aged 16	432 (19.8)	741 (22.1)	1,173 (22.1)
Aged 17	314 (14.4)	502 (15.3)	816 (15.4)
Location			
Rural	1,055 (48.5)	1,739 (55.4)	2,794 (52.6)
Urban	1,122 (51.5)	1,399 (44.6)	2,521 (47.4)
Living with			
Parents	1,997 (91.5)	2,892 (92.2)	4,889 (92.0)
Single parent	170 (7.8)	235 (7.5)	405 (7.6)
Neither with mother/father	10 (0.5)	11 (0.3)	21 (0.4)
Living in a family that experienced difficulty buying food	548 (25.2)	905 (28.8)	1,453 (27.3)
Having at least one parent unemployed due to COVID	442 (20.3)	850 (27.1)	1,292 (24.3)
Experiencing domestic family violence during the COVID time	218 (10.0)	401 (12.8)	619 (11.6)
Average Online time per day			
Less than 4 h	987 (45.3)	1,241 (39.5)	2,228 (41.9)
4 to 6 h	182 (8.4)	260 (8.3)	442 (8.3)
6 to 8 h	475 (21.8)	708 (22.6)	1,183 (22.3)
8 h or more	533 (24.5)	929 (29.6)	1,462 (27.5)

Table 2 shows that more than one-third (37.2%) of the participants had parents or guardians who often or always check their homework. According to nearly half of the participants, their parents or guardians often or always understood their problems/worries during the past 30 days. Moreover, half of the children reported that their parents or guardians knew what they did in their free time, and 53.5% often or always received advice and guidance from their parents or guardians during the past 30 days.

**Table 2:** Family care and family pressure during the COVID-19 pandemic.

**Family care and family pressure during the COVID-19 pandemic**	**Frequency**				
	**Never *n* (%)**	**Rarely *n* (%)**	**Sometimes *n* (%)**	**Often *n* (%)**	**Always *n* (%)**
**Family care (Mean: 9.5; sd: 3.0)**					
The parent/guardian checked if their child did homework during the past 30 days.	862 (16.2)	606 (11.4)	1,868 (35.1)	1,425 (26.8)	554 (10.4)
The parent/guardian understood their child's problems and worries during the past 30 days.	520 (9.8)	829 (15.6)	1,502 (28.3)	1,125 (21.2)	1,339 (25.2)
The parent/guardian knew what their child was doing in his/her free time during the past 30 days.	412 (7.8)	584 (11.0)	1,677 (31.6)	1,158 (21.8)	1,484 (27.9)
The parent/guardian gave their child advice and guidance during the past 30 days.	230 (4.3)	455 (8.6)	1,786 (33.6)	1,409 (26.5)	1,435 (27.0)
**Family pressure (Mean: 4.8; sd: 2.1)**					
The parent/guardian expected too much of their child (i.e., to perform better at school or to be a better person) during the past 30 days.	321 (6.0)	441 (8.3)	1,317 (24.8)	2,012 (37.9)	1,224 (23.0)
The parent/guardian did not respect their child as a person (i.e., not letting their child talk or favoring someone else more than their child) during the past 30 days.	2,824 (53.1)	956 (18.0)	355 (6.7)	427 (8.0)	753 (14.2)
The participant (i.e., the child) was involved in a physical fight during the past 12 months.	592 (11.1)	4,402 (82.8)	31 (0.6)	30 (0.6)	260 (4.9)

Regarding family pressure (Table 2), 60.9% of participants reported that their parents or guardians often or always expected too much of them. Nearly one-fourth of parents or guardians often did not respect their child as a person during the past 30 days. Children who reported being often and always involved in a physical fight during the past 12 months accounted for 5.5%.

Table 3 shows the respondents' self-reports of how they confronted a stressful situation during the COVID-19 pandemic. In the three groups of active coping strategies, “Saying sorry if it is your fault” was the most applied (57.4%), followed by “Thinking about what others might do” (50.6%), and “Trying your best to make things better” (48.6%). Only 3.8% reported trying hard to fix the problems they encountered. The average total score of coping was 20.40 (std = 2.13).

**Table 3:** Self–reports of coping with a stressful situation during the COVID-19 pandemic.

**Kid cope scale**	**Never; *n* (%)**	**Sometimes; *n* (%)**	**Always; *n* (%)**
**Problem-solving**			
Trying to think of a different way to solve the problem	138 (2.6)	3,654 (68.7)	1,523 (28.7)
Trying your best to make things better	93 (1.7)	2,640 (49.7)	2,582 (48.6)
Trying hard to fix the problem	1,785 (33.6)	3,329 (62.6)	201 (3.8)
Saying sorry if it is your fault	71 (1.3)	2,194 (41.3)	3,050 (57.4)
**Avoidant**			
Distracting yourself from thinking about the problem	67 (12.7)	3,753 (70.6)	886 (16.7)
Avoiding the problem or the area where it happened	160 (3.0)	2,674 (50.3)	2,481 (46.7)
Doing something else to stop thinking about the problem	888 (16.7)	2,616 (49.2)	1,811 (34.1)
**Social support seeking**			
Thinking about what others might do	717 (13.5)	1,907 (35.9)	2,691 (50.6)
Seeking help from others	264 (5.0)	2,965 (55.8)	2,086 (39.2)

**Figure 1:**
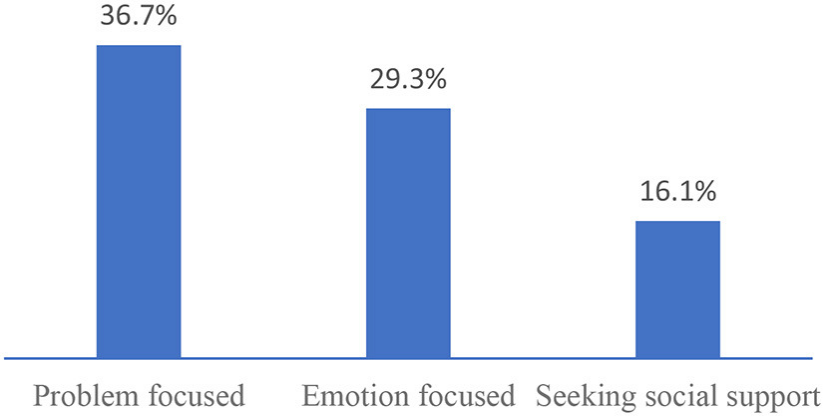
The percentage of adolescents having issues with the coping domain.

Figure 1 shows that the percentage of adolescents having issues with emotion-focused coping, problem-focused coping, and seeking social support was 25.0, 32.9, and 32.8%, respectively.

Table 4 shows that daily internet usage was negatively associated with the children's coping score (*p* < 0.001) in the multivariate model, with other factors being held constant. Staying online for about 6 to 8 h per day or at least 8 h led to a 0.45-fold decrease (95% CI: −0.29; 0.61) and a 0.62-fold decrease (95% CI: 0.47; 0.78) in the general coping score, respectively.

**Table 4:** Regression model for the relationship of internet use duration with the coping of the children.

**Characteristics**	**Univariate analysis**		**Multivariate analysis**	
	**Coeff**	**95%CI**	**Coeff**	**95%CI**
**Internet use duration per day**				
Less than 4 h	Ref		Ref	
4 to under 6 h	−0.17	−0.4 to 0.06	−0.13	−0.36 to 0.09
6 to under 8 h	−0.61^***^	−0.77 to −0.45	−0.45^***^	−0.61 to −0.29
8 h and more	−0.86^***^	−1.01 to−0.72	−0.62^***^	−0.78 to −0.47
**Gender**				
Male	Ref		Ref	
Female	−0.07	−0.19 to 0.06	0.01	−0.11 to 0.13
**Location**				
Urban	Ref		Ref	
Rural	0.02	−0.04 to 0.09	0.06^*^	0.001 to 0.12
**Living with**				
Parents	Ref		Ref	
Single parent	0.07	−0.16 to 0.3	0.13	−0.09 to 0.36
No one	−1.31^**^	−2.28 to −0.33	−1.02^*^	−1.97 to −0.06
Family care	0.14^***^	0.12 to 0.16	0.11^***^	0.08 to 0.13
Family pressure	−0.04^*^	−0.07 to −0.01	−0.02	−0.05 to 0.01
Having at least one parent unemployed due to COVID.	0.25^***^	0.11 to 0.39	0.2^**^	0.06 to 0.34
Living in a family that experienced difficulty buying food	0.26^***^	0.12 to 0.4	0.25^***^	0.11 to 0.38
Experiencing domestic family violence during the COVID time	−0.46^***^	−0.65 to−0.27	−0.17	−0.37 to 0.03

^*^p < 0.05, ^**^p < 0.01, ^***^p < 0.001.

A 1-unit increase in covariate corresponds to a b-increase in the total coping score (if the coefficient >0) or a b-decrease in a total coping score (if the coefficient <0).

In addition, living in a rural area, having family care, having at least one parent unemployed due to COVID, living in a family that experienced difficulty buying food, and living alone were factors related to children's coping score (*p* < 0.05). Meanwhile, gender, family pressure, and experience of domestic family violence during COVID time had no significant association with their coping score (*p* > 0.05), with other factors being held constant.

## Discussion

### Kid coping

The present study reports on adolescents coping with challenging situations during the COVID-19 pandemic in Hanoi, Vietnam. Research on coping and emotion regulation in children and adolescents is critical for the field of developmental psychopathology and prevention science because of its potential to inform our understanding of risk, resilience, and intervention processes. Our findings from the analysis suggest that kid coping of children in Hanoi was quite low.

Coping strategies are related to symptoms of psychopathology (12). Avoidant coping is a risk factor for developing anxiety or depression in adolescents (7, 23, 24). In contrast, children with active coping, including problem-solving, predicted a lower risk of new-onset depression later (25–27). Adolescents who seek social support are less likely to develop depression and anxiety symptoms (12, 15, 28).

In our study, about 87% of adolescents reported “distracting yourself from thinking about the problem” this result was similar to the study of Campbell et al. (29), with 84.7% of adolescents reported “just tried to forget it” when they faced with a problem. There was 83.3% of adolescents reported “doing something else to stop thinking about the problem,” and 66.4% of adolescents reported “trying hard to fix the problem” those results were higher than the results of Campbell et al. (29). The reason may be due to our research conducted in the context of the Covid-19 pandemic, and the culture in diffentce.

In line with the previous studies, children utilized a variety of strategies to cope with stress (28–31), reflecting a need for children to attempt to cope. Children most frequently reported using avoidant coping and social coping, findings that are consistent with the existing research (29, 30). Children do not use a single strategy to cope, supporting speculation presented by other studíe that children essentially “try out” a large variety of strategies before determining which are effective and/or feasible (28–30, 32, 33).

Although our study did not address children's assessment of effective coping strategies, many studies show that social support or emotional coping methods were partially effective and better than avoidance strategy (29–31, 33, 34). Zimmer et al. also pointed out that proactive coping, not avoidant, was related to better psychological well-being in children, but only in controllable situations/contexts (13). Future research should consider the effectiveness of coping strategies in adolescents.

Several adolescents still hide/avoidant the problems they face instead of thinking positively and solving them. This is dangerous because youths who engage in avoidant coping behaviors (such as distraction, self-blame, or behavioral disengagement) are more likely to experience emotional distress. In contrast, those who engage in active/adaptive coping strategies are less likely to experience negative mental health symptoms (24, 35, 36). However, in uncontrollable situations, for example, the COVID-19 pandemic, the secondary control strategies (i.e., avoidant) may be more effective (13, 37). Because of the global scale of the COVID-19 spread, young people may feel powerless to change the situation, resorting to secondary control strategies such as distraction to deal with the pandemic's effects.

### Relationship between internet use and kid coping

Bach et al. indicated that COVID-19 restrictions increased the global internet use (38). The change in children's Internet use and their activities on the internet also directly affects how children face and deal with other risks. Internet use has been linked to loneliness and can provide short-term relief and relief from stress in response to the psychological distress of the pandemic. The literature also suggests increased Internet use is associated with poor psychological regulation (39).

The study results showed that the coping score decreased as the internet use duration increased. This result corresponds to Singh et al. (40); with increased internet usage, there was an increase in maladapted coping. This can be explained by the fact that children spend much time on the internet, so they do not have time to think and actively face stressful issues in life. The virtual world created by the internet offers children the opportunity to escape external stress and real-life difficulties through short-term joys and relief. However, this also reduces the child's ability to cope with reality and will affect the child's mental health capacity, making the child use the internet more and leading to internet addiction.

### Strength and limitation

This study has some important strengths. As already underlined, this is one of the first studies conducted in Vietnam about children coping with stress and the relationship between internet use duration and the coping of the children. Another strength is the large sample size, with more than 5,000 Hanoian teenagers participating in our study.

Regarding limitations, and due to the characteristics of this current time of isolation, all the respondents answered an online questionnaire using self-reported measures; hence, the accuracy of the answers and the potential influence of self-report bias on the results could not be determined. Second, a precise causal relationship between the variables cannot be established because this is a cross-sectional study. Third, the respondent-driven sampling technique was another limitation; the sampling process was non-random and could lead to potential sampling bias. Future research should employ face-to-face self-administered interviews and a probability sampling approach to select more representative respondents.

## Conclusion

During the COVID-19 pandemic, Hanoi adolescents with a score of coping skills were moderate. Many of them use the internet for 8 h and more per day. Internet use has an adverse impact on their coping. Therefore, parents and family members should provide a supportive environment, which may improve adolescents' coping skills and, as a result, lower their risk of mental health. The future study should determine the effectiveness of coping strategies in adolescents and children's coping skills from a parent's perspective.

## Data availability statement

The raw data supporting the conclusions of this article will be made available by the authors, without undue reservation.

## Ethics statement

Ethical clearance, including confidentiality of the participants' consents and information, was approved by the Human Research Ethics Committee at Hanoi University of Public Health. No sensitive data that could identify the participants was collected. Informed consent was described in the instrument, with mandatory acceptance to proceed. There was also a contact for further clarification. Written informed consent to participate in this study was provided by the participants' legal guardian/next of kin.

## Author contributions

QB and DP designed and conceptualized the paper. QB and CP analyzed the data. All authors interpreted the results, prepared and reviewed the manuscript, and contributed to the critical revision of the manuscript for important intellectual content read and approved the final manuscript.

## Conflict of interest

The authors declare that the research was conducted in the absence of any commercial or financial relationships that could be construed as a potential conflict of interest.

## Publisher's note

All claims expressed in this article are solely those of the authors and do not necessarily represent those of their affiliated organizations, or those of the publisher, the editors and the reviewers. Any product that may be evaluated in this article, or claim that may be made by its manufacturer, is not guaranteed or endorsed by the publisher.
